# 

**Published:** 1917-04-21

**Authors:** 


### CONTENTS.

| | PAGE |
|---|---|
| **Pain and Shock** ... ... ... ... ... ... ... | 39 |
| **An Attack on the Army Medical Service** ... | 40 |
| **Hospital and Institutional News** ... ... ... | 41 |
| The Outrages on Hospital Ships—Death of a late Director-General of the Army Medical Service—The Rank of Consultants in the R.A.M.C.—Two Craftsmen and Queen Alexandra—The Huns Surpass Themselves—Marriage of Sir W. J. Thomas—The Two Perils to Soldiers' Pensions—The Walker Memorial at Peterborough—A Special Appeal for the Liverpool Royal Southern Hospital—The Works Department at Guy's—Infant Welfare Work: The New Phase—Should Salaries be Published in Detail?—Accidents to Patients in a Dundee Institution—School Buildings Converted to Hospital—A Flourishing County Nursing Association—Women Doctors Farm for Disabled Soldiers—A Fine Achievement—This Week's Drug Market. | |
| **War and Eugenics** ... ... ... ... ... ... | 45 |
| A Paroxysm of Paradox. | |
| **A Ministry of Health** ... ... ... ... ... ... | 46 |
| **Voluntary Hospital Finance** ... ... ... ... | 47 |
| I. Some Reflections and a Foreword. | |
| **Legal Notes for Institutional Workers** ... ... | 48 |
| **A Foreword, with Some Hospital Reminiscences** | 49 |
| **The Bedford County Hospital** ... ... ... ... | 52 |
| The Guild of Social Workers. | |
| **The Matrons' and Sisters' Department** ... | 53 |
| The Profession of British Nurses: Plain Words on Registration.—IV. Noble Women Workers in the War. The Devotion of Queen Alexandra—Noble Nurses in the Homeland—A Concert for Nurses—When Nurse Training Commenced—Night Nursing Disapproved—A Tale of Sister Dora. | |
| **Food in Wartime** ... ... ... ... ... ... | 55 |
| Bread as the Staple Food. | |
| **Labour-Saving Appliances** ... ... ... ... | 56 |
| **The Editor's Letter-Box** ... ... ... ... | 57 |
| **The Institutional Worker** ... ... (Suppl.) | 1 |

